# Supplementary material for: Temperate Lianas Have More Acquisitive Strategies than Host Trees in Leaf and Stem Traits, but Not Root Traits
Source: Plants (Basel). 2022 Dec 15;11(24):3543. doi: 10.3390/plants11243543 (PMC9788099; doi:10.3390/plants11243543)
Supplement: Supplementary file 1 [file plants-11-03543-s001.zip › plants-2070361-supplementary.pdf]

Supplementary Materials

Table S1. Lianas and host tree species sampled in this study (BD: basal diameter, cm, DBH: Diameter at breast height, cm).

Lianas			Host trees		
**Species**	**Family**	**BD**	**Species**	**Family**	**DBH**
Actinidia chinensis	Actinidiaceae	8.1	*Quercus aliena var. acutiserrata*	Fagaceae	16.4
		5.9	*Celtis koraiensis*	Cannabaceae	16.6
		5.4	*Acer davidii*	Sapindaceae	12.1
		7.5	*Acer davidii*	Sapindaceae	20.4
		4.8	*Quercus aliena var. acutiserrata*	Fagaceae	26.8
		7.8	*Quercus aliena var. acutiserrata*	Fagaceae	25.1
		2.7	*Acer stachyophyllum*	Sapindaceae	13.0
Akebia trifoliata	Lardizabalaceae	4.1	*Quercus aliena var. acutiserrata*	Fagaceae	14.5
		2.5	*Quercus aliena var. acutiserrata*	Fagaceae	10.2
		4.5	*Quercus aliena var. acutiserrata*	Fagaceae	16.7
		3.0	*Quercus aliena var. acutiserrata*	Fagaceae	30.4
		1.5	*Quercus aliena var. acutiserrata*	Fagaceae	8.2
		2.1	*Quercus aliena var. acutiserrata*	Fagaceae	7.3
		3.5	*Quercus aliena var. acutiserrata*	Fagaceae	18.0
		3.2	*Quercus aliena var. acutiserrata*	Fagaceae	18.5
		3.2	*Quercus aliena var. acutiserrata*	Fagaceae	31.7
		2.1	*Quercus aliena var. acutiserrata*	Fagaceae	14.1
		3.2	*Acer davidii*	Sapindaceae	10.7
		3.5	*Quercus aliena var. acutiserrata*	Fagaceae	15.7
		2.4	*Quercus aliena var. acutiserrata*	Fagaceae	9.5
		2.9	*Quercus aliena var. acutiserrata*	Fagaceae	27.0
		0.8	*Acer davidii*	Sapindaceae	3.9
Celastrushypoleucus	Celastraceae	13.0	*Lindera obtusiloba*	Lauraceae	27.0
		3.0	*Quercus aliena var. acutiserrata*	Fagaceae	18.6
Celastrushypoleucus	Celastraceae	6.8	*Quercus aliena var. acutiserrata*	Fagaceae	14.5
		8.4	*Acer davidii*	Sapindaceae	26.8
		4.3	*Acer davidii*	Sapindaceae	20.7

		4.8	*Quercus aliena var. acutiserrata*	Fagaceae	22.8
		6.4	*Quercus aliena var. acutiserrata*	Fagaceae	40.2
		5.2	*Quercus aliena var. acutiserrata*	Fagaceae	33.9
		3.4	*Quercus aliena var. acutiserrata*	Fagaceae	21.4
		5.2	*Quercus aliena var. acutiserrata*	Fagaceae	26.5
		6.2	*Lindera obtusiloba*	Lauraceae	14.8
		3.9	*Quercus mongolica*	Fagaceae	20.0
		5.4	*Salix matsudana*	Salicaceae	32.0
		5.3	*Betula albosinensis*	Betulaceae	30.1
		4.2	*Betula albosinensis*	Betulaceae	18.0
		1.1	*Populus purdomii*	Salicaceae	40.0
Schisandra sphenanthera	Schisandraceae	4.5	*Quercus aliena var. acutiserrata*	Fagaceae	31.3
		3.5	*Quercus aliena var. acutiserrata*	Fagaceae	30.8
		5.3	*Quercus aliena var. acutiserrata*	Fagaceae	39.1
		3.8	*Quercus aliena var. acutiserrata*	Fagaceae	33.0
		3.5	*Quercus aliena var. acutiserrata*	Fagaceae	13.4
		5.6	*Quercus aliena var. acutiserrata*	Fagaceae	37.9
		4.9	*Quercus aliena var. acutiserrata*	Fagaceae	37.6
		2.6	*Acer davidii*	Sapindaceae	13.9
		2.8	*Quercus aliena var. acutiserrata*	Fagaceae	23.3
		4.3	*Quercus aliena var. acutiserrata*	Fagaceae	22.0
		3.5	*Quercus aliena var. acutiserrata*	Fagaceae	30.8
		5.6	*Populus purdomii*	Salicaceae	31.0
		3.5	*Quercus aliena var. acutiserrata*	Fagaceae	21.9
		3.8	*Quercus aliena var. acutiserrata*	Fagaceae	26.0
		3.9	*Acer davidii*	Sapindaceae	17.8
		2.6	*Quercus aliena var. acutiserrata*	Fagaceae	42.6
		9.7	*Quercus aliena var. acutiserrata*	Fagaceae	29.2
Vitis piasezkii	Vitaceae	9.2	*Padus avium*	Rosaceae	23.6
		4.3	*Fraxinus chinensis*	Oleaceae	13.4
		3.5	*Salix matsudana*	Salicaceae	51.0
		5.1	*Prunus polytricha*	Rosaceae	14.0
		5.3	*Salix matsudana*	Salicaceae	25.0
		2.9	*Populus purdomii*	Salicaceae	25.2

Table S2. Differences in slope and shifts in elevation (intercept) between all sampled lianas (*n* = 61) and trees (*n* = 61).

Traits		Slope		Elevation	
X	Y	Lianas	Trees	Lianas	Trees
L_{th}	LA	1.92	−3.43	4.97	−4.63
L_{th}	LDMC	−1.09	0.52	−2.53	0.59
L_{th}	LCC	0.24	−0.34	3.01	1.99
L_{th}	WD	−0.51	0.61	−3.12	−0.98
L_{th}	SRL	−0.72	−1.47	1.01	−0.5
LA	LDMC	−0.57	−0.15	0.26	0.12
LA	LNC	0.27	0.27	**0.97**	**0.95**
LA	LCC	0.12	0.11	2.4	2.45
LA	WD	0.26	0.18	−2.64	−2.47
LDMC	LMA	0.43	1.96	−2.11	−1.49
LDMC	SLA	**−0.44**	**−1.95**	2.11	1.31
LDMC	LNC	0.48	−1.79	1.69	0.73
LDMC	LCC	0.22	0.65	2.73	2.9
LDMC	WD	0.46	1.15	−1.95	−1.66
LMA	SLA	−1.02	−0.99	−0.04	0.01
LMA	LNC	1.12	−0.91	4.05	−0.63
LMA	LCC	0.5	−0.33	3.78	1.89
LMA	WD	1.08	−0.59	0.33	−3.48
SLA	LNC	−1.1	0.92	4.01	−0.64
SLA	LCC	−0.49	−0.33	3.76	1.88
SLA	WD	−1.06	0.59	0.29	−3.48
SLA	RCC	−0.46	−0.49	3.71	3.75
LNC	LCC	0.45	0.36	**1.97**	**2.11**
LNC	WD	**0.96**	**0.65**	−3.58	−3.3
LNC	RNC	−1.37	−1.57	3.17	3.54
LCC	WD	2.16	1.79	−7.82	−6.84
RNC	RCC	0.31	0.34	2.23	2.2

Note: Significant differences (P < 0.05) are indicated by the superscripts and marked in bold. For explanation of abbreviations, see Table 1.

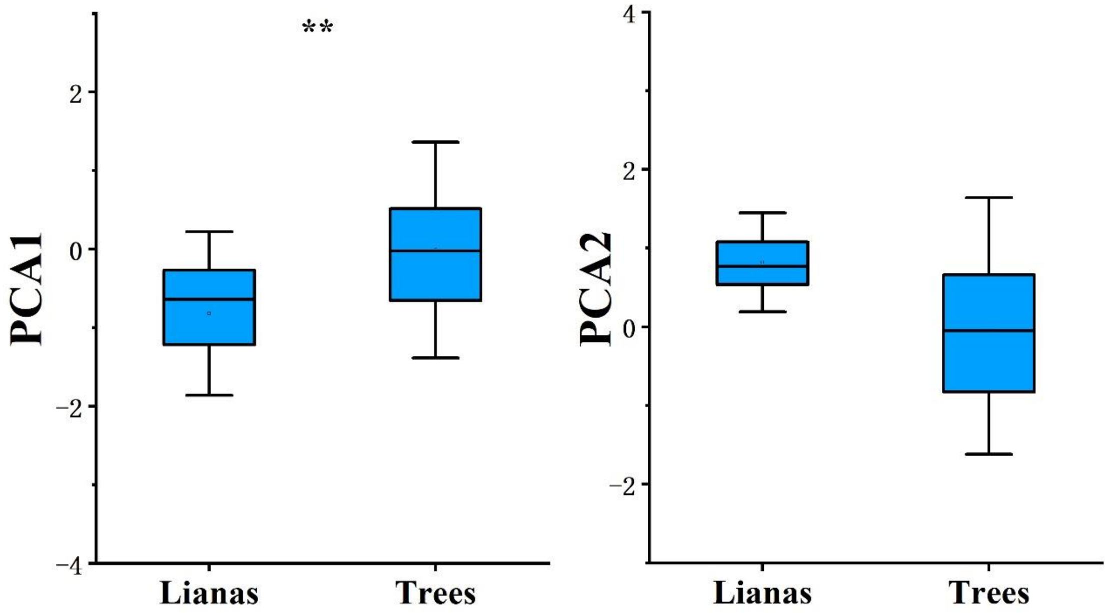

Figure S1. Differences between lianas and host trees in the first and second component scores for the forest. Asterisks indicate statistically significant difference between lianas and host trees at $\alpha < 0.001$.
